# Illness beliefs and emotional responses in mildly disabled stroke survivors: A qualitative study

**DOI:** 10.1371/journal.pone.0223681

**Published:** 2019-10-23

**Authors:** Claire Della Vecchia, Marie Préau, Camille Carpentier, Marie Viprey, Julie Haesebaert, Anne Termoz, Alexandra L. Dima, Anne-Marie Schott

**Affiliations:** 1 Univ. Lyon, Université Claude Bernard Lyon 1, HESPER EA 7425, Lyon, France; 2 GRePS Lyon 2 Université, University of Lyon, Bron, France; 3 Aix-Marseille Université, INSERM UMR 912 SESSTIM, Marseille, France; 4 Hospices Civils de Lyon, Pôle Santé Publique, Lyon, France; University of Wisconsin Madison School of Pharmacy, UNITED STATES

## Abstract

**Background:**

As acute stroke services improve, more persons experience mild stroke and need to cope daily with hidden disabilities, which may be influenced by how they perceive stroke, cognitively and emotionally.

**Objective:**

To investigate cognitive illness beliefs and emotional responses in persons with mild stroke and their possible influences on daily coping.

**Methods:**

Semi-structured interviews were conducted with 24 persons with mild stroke, on average 7.5 months (±0.89) after stroke occurrence. A thematic analysis on verbatim transcripts was guided by the Common-Sense Model of Self-Regulation.

**Results:**

All participants experienced difficulties constructing an illness identity at both acute and chronic phase. Behavioral risk factors were less accepted as causes of stroke. Lack (or inappropriate timing) of information from healthcare providers led to limited medication knowledge and low perceived control of stroke recurrence which generated anxiety, fear, and low involvement in coping. Participants who considered stroke a chronic condition experienced more difficulties. Perceived support from relatives and healthcare providers was beneficial for participation in recovery and health behaviour change.

**Conclusion:**

Despite having mildly disabilities, participants reported difficulties developing illness beliefs conducive to coping, and dealing with their emotional responses. These elements should be considered in tailored programs to improve coping with hidden disabilities post-stroke.

## Introduction

Fifty million survivors of stroke live with post-stroke disability worldwide; this includes both visible and hidden disabilities [1]. Research on the concept of hidden disability is in its early stages, and a clear definition and classification is yet to be agreed upon. Recent definitions include fatigue both mental and physical, impaired concentration ability, memory difficulties, irritability and emotional problems (emotional lability, anxiety, depression) as examples of such disabilities [2,3]. The term Astheno-Emotional Disorder (AED) has also been proposed, and defined by “mental fatigability, concentration difficulties, memory difficulties, irritability and emotional instability, sensitivity to bright light and loud sounds and stress sensitivity” [4,5]. These hidden consequences may be associated with more visible disabilities (physical disabilities) or may occur independently in persons with mild stroke. Mild strokes have been defined in various ways: a National Institute of Health Stroke Scale (NIHSS) score <6 [5], a modified Rankin scale ≤ 2 [6] (“Slight disability; unable to carry out all previous activities, but able to look after own affairs without assistance”) [7]), or an absence of significant aphasia, unilateral spatial neglect or major motor problems, and a capacity to manage all basic activities of daily living [8]. They may represent up to 50% of survivors of stroke [8].

Due to limited resources, not all persons with stroke benefit from rehabilitation services after their acute care hospitalization. In France for example, only 30% of persons with stroke have access to a rehabilitation center [9]. Persons mildly disabled are likely to return home directly after acute hospitalization. They may have an appointment with a specialist several months after their stroke, but their medical follow-up is mostly performed by general practitioners who may not have specific training for the identification and management of hidden disabilities.

There is limited research on the impact of mildly disabling stroke on survivors’ daily lives. From the few existing studies, they have been associated with a higher risk of developing mental health problems, experiencing difficulties with complex activities in daily life and a decrease of self-management ability all impacting negatively adoption of healthy behaviors, adherence to secondary stroke prevention, and eventually stroke recurrence[5,10].

Consequently, there is a need to better understand the way mildly disabled persons cope with stroke. This could help community services and healthcare providers better target the support they provide to survivors’ needs and thus facilitate self-management of stroke and its consequences.

**Fig 1:**
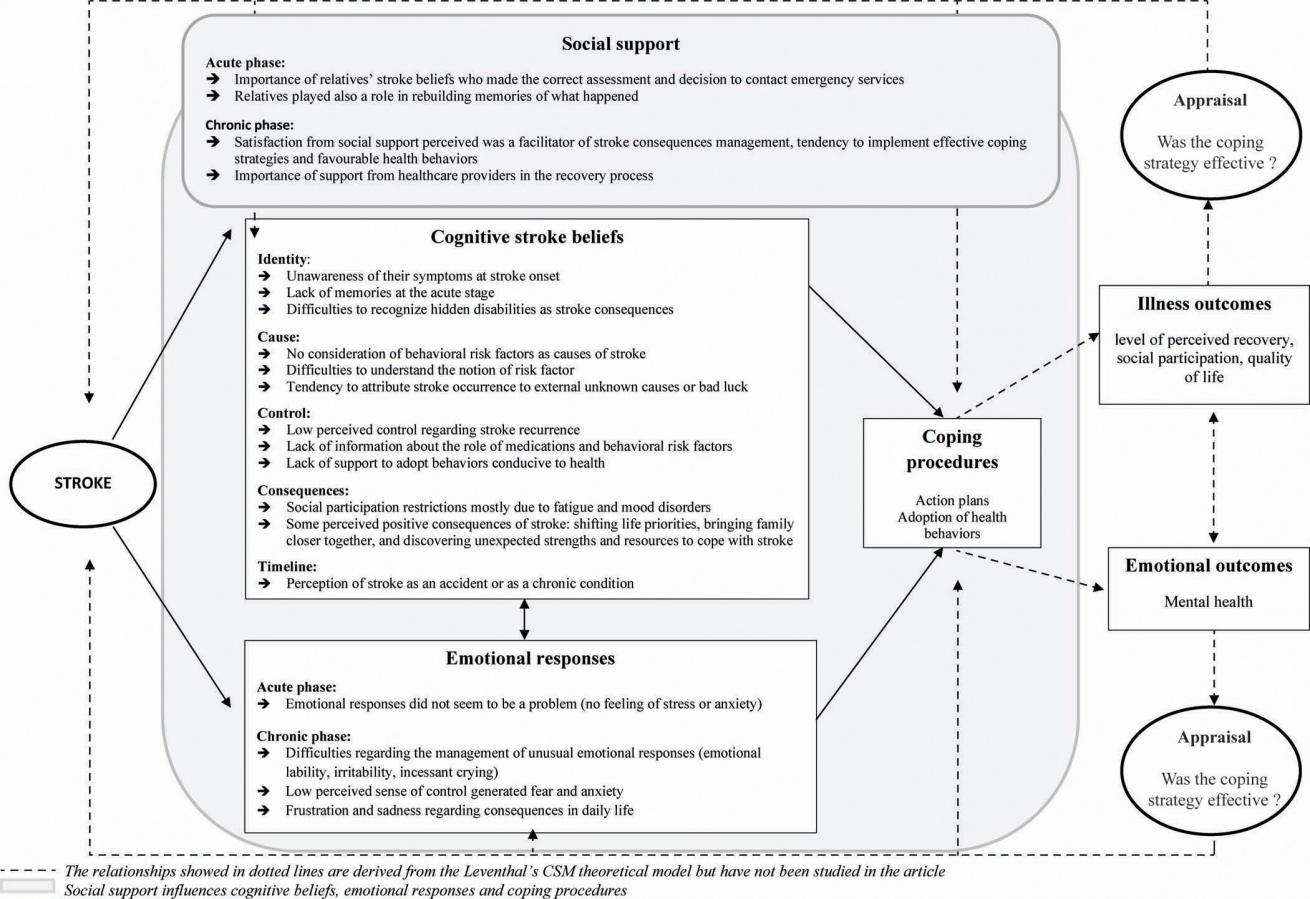
The stroke common sense model of self-regulation adapted from Leventhal et al, 2016 [11].

A framework that has proven useful for understanding how individuals cope with health problems is the Common-Sense Model of Self-Regulation (CSM) (Fig 1) [11].

According to this model, illness can be experienced as a threat, in relation to which individuals develop cognitive illness beliefs and emotional responses that will in turn determine their decisions on how to best cope with the current symptoms (e.g. change their health behaviors, follow the prescribed treatment)[11,12]. Illness beliefs and their impact on the way people cope with illness have been investigated in various chronic conditions such as diabetes, irritable bowel syndrome, asthma, hypertension and cancer [12–14] using mainly quantitative methods especially the Illness Perception Questionnaire (IPQ) [15], its revised version [16] or its brief version [17]. In persons suffering from stroke, it has been found that willingness to adopt behaviors conducive to health depends on their illness beliefs [18].

There exists only limited research on survivors’ cognitive illness beliefs and emotional responses after mild stroke; we identified a single recent study which focused on beliefs about treatment adherence, adopted a quantitative methodology and generated limited data on how survivors perceive the condition itself cognitively and emotionally [19]. A comprehensive qualitative exploration of these representations as they are experienced by the persons would provide key information regarding their support needs after stroke occurrence. Our aim was therefore to investigate the illness beliefs and emotional responses of persons mildly disabled after stroke and their potential impact on the way they cope with stroke and its consequences. A better understanding of these aspects could help identify potential gaps in services and develop appropriate healthcare support and services to improve the quality of life of stroke survivors.

## Methods

We conducted a qualitative study using face-to-face semi-structured interviews. The study was reported using COREQ criteria [20] (S1 Table). According to French legislation, this study received approval from ethics committee (Comite de Protection des Personnes Sud Mediterranée III). All subjects interviewed gave written informed consent.

**Trial Registration:** This study has been registered at clinicaltrials.gov (TYBRA study, identifier: NCT03217279).

### Research design and methods

**Fig 2:**
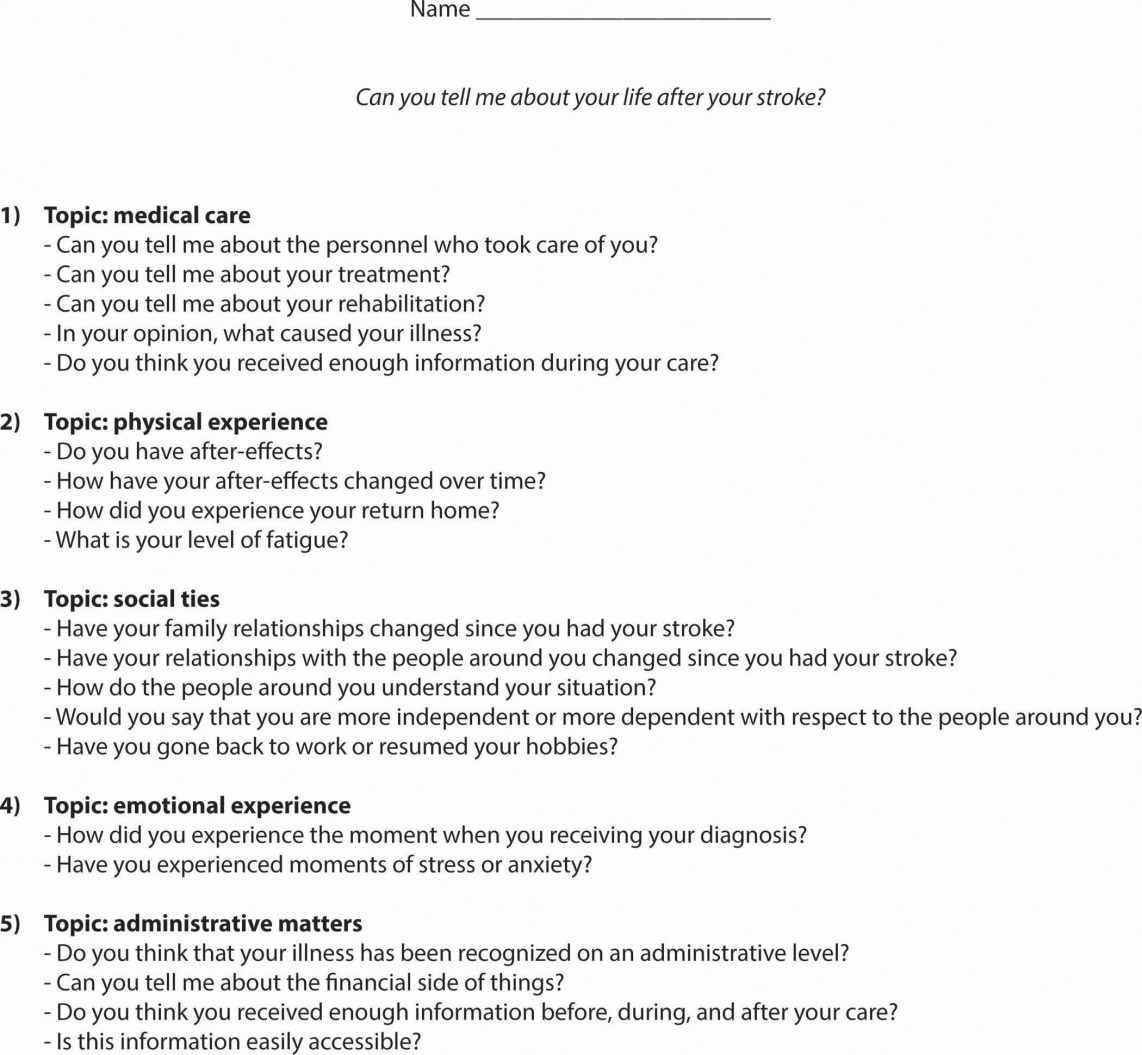
Semi-structured interview guide.

To conduct our qualitative study using face-to-face interviews we needed to develop a semi-structured interview guide. As we found limited prior research on the subject, we first performed several unstructured interviews to inform the development of the interview guide. By this initial step, we aimed to ensure that the guide allows up to capture the diversity of participants' concerns and beliefs. Two researchers (CDV and CC, PhD student in public health and in health psychology respectively) who were trained in qualitative methods conducted 8 unstructured interviews (not included in the final analysis). Based on an exploratory approach, these interviews began with a broad question about the experience of stroke and used rephrasing and probing questions to encourage participants to talk about different elements of their experience [21]. On the verbatim transcriptions of these initial interviews, a data-driven thematic analysis was conducted i.e. an inductive approach without the use of a theoretical framework to guide the analysis to ensure an exploratory approach in the construction of our semi-structured interview guide [22]. Thematic analysis consists in analyzing and identifying the recurring themes that structure the participants' discourses [22]. Five themes were identified in participants’ discourse: 1) medical care, 2) perceived disabilities and the management of the return home, 3) social support, 4) emotional responses to stroke, and 5) administrative procedures. These themes were used to develop a semi-structured interview guide for the second stage (Fig 2), in which CDV and FM (post-doctoral researcher in health psychology) conducted face-to-face semi-structured interviews to investigate participants’ beliefs of stroke and emotional responses at approximatively six months post-stroke. We included participants at approximatively six months after stroke from a population-based prospective cohort study that took place between November 2015 and June 2016 [23]. Participants were all persons aged over 18 years with an acute stroke admitted to any emergency department (ED) or stroke unit of the Rhône area of France (1.7 million inhabitants). Definitive stroke diagnosis was based on cerebral imaging (computed tomography scan or magnetic resonance imaging) and confirmed by a neurologist. Participants were eligible for the present study if they had a modified Rankin Scale (mRS) ≤ 2 (‘slight disability; unable to carry out all previous activities but able to look after own affairs’) [24] and were not institutionalized at six months post-stroke. Among potentially eligible participants, we used the maximum variation sampling method to select a wide range of participants’ socioeconomic and demographic profiles; we split the cohort into four subgroups based on age (<65 years old and ≥65 years old), sex and place of residence (urban or rural area) in men and similar four subgroups in women, and sampled eligible participants in each subgroup to ensure maximum variation in our sample [25]. We initially recruited 15 participants and after that, we recruited and interviewed the participant and recruited the next participant until data saturation, i.e. when no additional substantial differences were noted in participants’ discourse [26]. Eligible participants were invited to participate by telephone; when a person refused, we called the next person on the list with similar characteristics regarding age, sex, and place of residence. None of the researchers had prior knowledge of the participants. When study participation was proposed, we presented our research team, the study goals and modalities of participation. Participants were given both oral and written information about study participation (researchers’ contact details, aim of the study, and conditions of participation) and provided their written consent before being interviewed. Semi-structured interviews started with a broad question about the participant’s experiences of stroke, followed by open questions targeting the five themes. Socio-demographic data (age, sex, marital status, and employment status) and stroke-related information (date and type of stroke, and perceived sequelae) were collected at the end of the interview.

### Data analysis

Interviews conducted in the second stage were audiotaped and transcribed verbatim by CDV, FM, and MD. All identifying information was removed from the transcripts. The analysis was both data- and theory-driven. We initiated our thematic analysis using an inductive approach [22,27] and discussed preliminary results within our interdisciplinary research team. The team members were public health and health psychology researchers with complementary expertise in healthcare services, stroke care, qualitative research, social psychology and health behavior change. Discussions considered several theoretical models and their relevance for the participants’ discourse and selected the Common-Sense Model of Self-Regulation (CSM) [11] as the theoretical framework of analysis as corresponded to the largest proportion of the data. We therefore continued the thematic analysis using a deductive approach and focusing on participants’ cognitive beliefs and emotional responses of stroke [11].

The CSM framework is being increasingly used to study persons’ experiences of an illness and actions to manage their health [11]. It proposes five main types of cognitive illness beliefs: 1) which symptoms they consider to be part of the illness (Identity), 2) which causes, in their view, probably led to this illness (Cause), 3) how long they think their illness might last (Timeline), 4) what consequences they perceive on their lives (Consequences), and 5) whether they think the condition can be controlled with medication or own actions (Control). In addition to cognitive illness beliefs, persons may have emotional responses (emotional reactions generated by an illness) that would also require specific coping actions. Both cognitive illness beliefs and emotional responses guide individuals in their coping strategies to deal with a health threat, a process that will bear an impact on health-related outcomes (e.g., health status, quality of life, and medication adherence).

Participants’ discourse was deductively categorized according to the five domains of cognitive illness beliefs (identity, cause, consequences, timeline, and control) and the domain of emotional responses. In parallel, we used an inductive approach to identify other relevant themes. Coding was performed independently by two researchers (CDV and LL) who then compared results within each theme and sub-theme. The choice of themes, sub-themes and their interpretation were discussed by the two coders and a third researcher (AD). Intermediate and final results of the analysis were discussed with the broader research team. After coding all discourse relevant for stroke beliefs and emotional responses, we examined the results for theme saturation; we noted that each theme included sufficient examples of discourse to be considered for inclusion in the results, and discourse was homogeneous within the themes and subthemes identified, and presented clear differences across categories. We carefully highlighted participants’ experiences by ensuring that each theme was illustrated with relevant verbatim quotes.

## Results

Twenty-four interviews were included in the analysis. Data saturation was obtained after 24 interviews. We contacted 52 participants, 29 accepted to be interviewed, 3 were excluded because they were institutionalized in nursing homes at 6 months post-stroke, 1 due to presenting a severe communication impairment not detected during recruitment, and 1 because the caregiver was present throughout the interview, which potentially could introduce bias in participant’s discourse. Common reasons for refusal to participate were lack of time and reluctance to talk about stroke. Refusal was more frequent in women.

Interviews has a median timing of 7.6 months post-stroke (range 6.2–9.3 months), a mean duration of 54 minutes (range 32–93 minutes) and 21 were conducted at participants’ homes. Median age at stroke onset was 68.5 years (range 40–89 years), and 17 participants were male (Table 1). At the time of the interview, most participants were married or in a relationship (n = 15), five were widowed, two were divorced, and two were single. At stroke onset, 17 participants were retired and seven were employed. At 6 months post-stroke, 14 participants had an mRS score of 2, 5 participants a score of 1 and 5 participants a score of 0. Most participants reported both hidden disabilities and slight physical impairments. Nine participants were admitted to a rehabilitation center after the acute stage, 15 did not benefit from rehabilitation center services although they declared impairments that impacted their daily lives. Medical follow-up was in all participants carried out by their general practitioners (Table 2).

**Table 1:** Participants characteristics.

Respondent No.	Sex	Age (at stroke onset)	Time since stroke onset (months)	Marital status	Socio-professional category	Interview duration (minutes)
1	F	68	7.9	widowed	retired	51
2	M	68	6.2	married	retired	32
3	M	68	8.1	married	retired	47
4	M	72	6.6	widowed	retired	75
5	F	87	6.8	widowed	retired	69
6	M	51	7.2	in a relationship	active employment	55
7	M	70	8.9	married	retired	45
8	M	82	8.9	married	retired	40
9	M	53	9.3	married	active employment	93
10	F	46	8.6	single	active employment	76
11	M	81	6.9	married	retired	34
12	F	64	6.5	widowed	active employment	54
13	F	66	6.6	divorced	retired	77
14	M	58	7.3	divorced	active employment	56
15	M	72	7.3	married	retired	56
16	M	66	7.9	married	retired	55
17	M	71	7.8	married	retired	35
18	F	40	7.0	single	active employment	37
19	M	73	6.9	married	retired	40
20	M	76	7.9	married	retired	45
21	M	49	7.2	married	active employment	50
22	F	89	8.3	widowed	retired	50
23	M	69	8.6	married	retired	62
24	M	72	8.3	married	retired	48

**Table 2:** Clinical characteristics of study participants.

Respondent No.	mRs (evaluated from researchers)	Impairments declared by participants (at interview time)	Admission to a rehabilitation center	Outpatient rehabilitation	Healthcare providers with whom participants had current interactions
**1**	0	Memory loss, visual disorders	No	None	GP, cardiologist
**2**	2	Balance and motor disorders, fatigue, irritability	Yes	Physiotherapy	GP, cardiologist
**3**	2	Motor disorders, urinary incontinence, fatigue, visual disorders	Yes	None	GP, urologist
**4**	1	Pain, fatigue, balance and mood disorders	Yes	Physiotherapy	GP, cardiologist, neurologist
**5**	2	Fatigue, balance and mood disorders, disorientation, memory loss	No	None	GP
**6**	0	None	No	None	GP, cardiologist
**7**	0	Fatigue, mood disorders, irritability	No	None	GP, cardiologist, neurologist
**8**	1	None	No	None	GP, cardiologist, pneumologist
**9**	1	Sensitivity disorders, fatigue	Yes	Physiotherapy	GP, cardiologist
**10**	2	Fatigue, mood and concentration disorders	Yes	Physiotherapy, speech therapy	GP, neurologist, psychiatrist
**11**	2	Distal sensitivity and mood disorders	No	None	GP, cardiologist
**12**	2	Fatigue, visual and mood disorders	No	Physiotherapy	GP, ophthalmologist
**13**	2	Visual, mood, concentration disorders, fatigue, memory loss	No	None	GP, psychiatrist, ophthalmologist
**14**	2	Motor, balance, visual, mood disorders, fatigue	No	Physiotherapy	GP, neurologist
**15**	1	Fatigue, mood disorders	Yes	Physiotherapy^1^, speech therapy^1^	GP, cardiologist, neurologist
**16**	2	Concentration disorders, libido and sexual dysfunction, emotional lability, fatigue, irritability	No	Speech therapy^1^	GP, cardiologist, neurologist
**17**	0	Dyspraxia, fatigue	Yes	Physiotherapy^1^, speech therapy^1^	GP, endocrinologist
**18**	2	Vertigo, fatigue, mood disorders	No	None	GP, neurologist
**19**	1	None	No	None	GP, cardiologist, pneumologist
**20**	2	Balance disorders	No	Physiotherapy	GP, neurologist
**21**	2	Dysgraphia	No	Physiotherapy, speech therapy	GP, cardiologist
**22**	2	Balance disorders, paresthesia	Yes	None	GP, neurologist
**23**	2	Fatigue, balance disorders	No	None	GP
**24**	0	Dysgraphia, fatigue	Yes	Physiotherapy, speech therapy	GP, neurologist

^1^No more rehabilitation sessions at the time of the interview

The results of the CSM framework analysis are presented below for each dimension and summarized in Fig 1.

### Identity

Within the CSM framework, the identity dimension was first addressed by participants when they talked about their experience of stroke onset. All participants reported being unaware of their symptoms and not perceiving the urgency of the situation, except participant 18 who wanted to warn her colleagues but could not because of speech impairment. None of them called emergency medical services by him/herself. Most did not link the symptoms they experienced with the label ‘stroke’ identity was not developed at this stage. Some participants called neighbors (participants 1, 13, and 22), others were contacted by relatives and discovered by chance (participants 10, and 14) or waited until the following day to visit their general practitioner (participant 5).

“I didn’t have the reflex to call the SAMU (emergency medical services) […] You lose your capabilities, I didn’t have this reflex, it was the next morning I said to myself: oooh, maybe after all I should go tell the doctor… I didn’t know it was a stroke." (Participant 5)

For the 18 participants who were not alone, caregivers (spouses) or co-workers (participant 18) called emergency medical services.

The identity dimension was also addressed when participants discussed their hospital stay during the acute phase and notably their lack of memories of this stage, which made it difficult to develop an illness identity around their experience of stroke.

“I absolutely don’t remember this stroke episode, there’s really a 48-hour black hole there, I have no idea what happened” (Participant 15)

Due to cognitive difficulties and lack of memories at the acute stage, participants reported difficulty developing a stroke identity and adopt healthy behaviors. Participant 12 put forward the importance of the time of information about lifestyle changes because she did not remember the information she received at the acute phase and then could not follow dietary advice.

“They tell you so many things at the hospital, we don’t feel very well […] Well, you forget a lot of things.” (Participant 12)

The role of relatives is essential in the construction of identity at this stage. Participant 7 highlighted the importance of his wife in rebuilding his memories of what had happened.

“I have no particular memory of the moment when I had my stroke […] My wife told me (what had happened)” (Participant 7)

Finally, the identity dimension appeared also when participants spoke of the chronic phase and reported difficulties knowing which symptoms and disabilities were direct consequences of stroke especially in participants who did not receive rehabilitation.

“maybe not everything should be blamed on stroke” (Participant 4)

Participants tended to underestimate cognitive impairments and other hidden disabilities as consequences of stroke and to rather attribute them to other causes such as aging or comorbidities. They tended to link their hidden impairments (particularly cognitive impairments and fatigue) to processes independent of stroke.

“little memory lapses but not dramatic ones […] but with aging you often have them anyway”

(Participant 1)

“somewhat reduced capacities, that’s for sure, but is it because of the stroke, is it because of cancer, I have no idea at all” (Participant 17)

### Cause

Participants looked for a single direct cause (such as atrial fibrillation or another medical condition) responsible for their stroke. They usually disagreed with healthcare providers regarding risk factors, particularly for those that are behavior dependent. They generally did not consider risk factors such as tobacco, alcohol, fat consumption, or body mass index as playing a role in their stroke occurrence.

“They blame it all on tobacco, alcohol, stress, er, it’s automatically, they blame it on just anything, it can play a role, I’m not saying the contrary, but I don’t think that, that this is everything, I think there’s something else” (Participant 6)

Instead, when no direct medical cause was identified, participants tended to attribute to external unknown causes or bad luck.

“It’s a matter of bad luck” (Participant 9)

### Consequences

Even though all participants had a mRS ≤ 2 and were autonomous in daily life, they all reported physical, psychological, and cognitive consequences that affected their leisure activities, working life, and social relationships, thus impacting their daily life and inducing participation restrictions.

“My level of fatigue, […] I can’t do the things I did before, there’re things I avoid doing”

(Consequences for leisure activities, Participant 2)

“I returned to work full-time, then I realized that I, I couldn’t manage, I didn’t manage to follow along, um… extremely tired” (Consequences for working life, Participant 18)

(speaking of relationships with others) “They are more difficult. I tolerate … people less, my wife must say I don’t tolerate them at all anymore. I have, I can’t put up with any comments anymore, none at all … I’m much more irritable. So it’s sometimes a bit more difficult. Er … Otherwise, well, I try to, I try but it’s not always easy … But I think that it’s mainly that, it’s with my nerves that I, I have trouble … controlling myself” (Consequences for social relationships, Participant 7)

Among participants who were employed prior to their stroke (N = 7), only two returned to work thanks to adjustments made to their job roles. Those who did not return to work explained it was mainly due to fatigue and concentration difficulties. Interestingly some participants reported positive consequences of stroke (participants 6, 9, and 21), primarily shifting life priorities (family and health first, work second), bringing family closer together, and discovering unexpected strengths and resources to cope with stroke and its consequences.

“if someone had told me that I’d experience anything like it one day… If someone had told me that I’d overcome it again… I would frankly have hesitated” (Participant 9)

“I find that it brought us a lot closer together, we told each other things that we’d never said before. And then I think that, well, I think that all of our priorities are in a bit of a different order now because, I had a close call, I think. Of course you see things differently” (Participant 9)

### Control

The control dimension was addressed at the acute phase, when participants stressed the importance of having more people among their relatives and the general population capable of recognizing stroke symptoms and reacting quickly to minimize disability. Participants 9 and 18 highlighted the importance of stroke prevention campaigns through which they were unable to react on their own at stroke onset.

“I think that also it’s good to warn, even, er, it’s a very simple thing as I was saying to my friends, even just by putting ads on TV about strokes, how to detect strokes, I think it’s important” (Participant 18)

Only 3 participants (1, 4, 12) reported having received advice during their acute hospital or rehabilitation stay regarding dietary habits. However, participant 4 considered she had good habits and did not intend to change even if they did not exactly correspond to the recommendations received by the nurse at the acute phase. Participant 12 did not follow the advice given by her dietician at the acute phase because she forgot that she had received it. Participant 1 was disappointed by the lack of information and support concerning diet.

For some participants, stroke was the trigger of successful behavior changes they initiated on their own.

“Before (stroke) I was a little lazy to get into town, I used to take the car, but now I'm walking. […] I have quit (smoking) since the first day of the stroke” (Participant 2)

“I stopped smoking, alcohol and the birth control pill” (Participant 10)

“Cigarettes are over, it's okay, it's over. […] Well, we've changed our whole life that we used to do before. […] So we stopped because it was a stressful lifestyle. Start in the morning at 4:45 and come home at 8:00 in the evening. […] “I told myself if anything happened, I'd stop smoking completely.”

(Participant 21)

However, participant 14 pointed out his difficulties to quit smoking and the lack of support from healthcare providers.

“(talking about quitting smoking) since the month of, uh, December, but it's hard.” (Participant 14)

Control was also addressed at the chronic stage in relation to taking medication. Some participants reported that taking their medication helped them to reinforce their sense of control regarding their condition. However most participants claimed they were taking their medications just because healthcare providers told them to do so. As such, these participants were not aware of the precise role of each drug, nor how it could be beneficial for controlling their condition. In particular, they had no sense of control and no action plans or strategies clearly implemented in daily life regarding the risk of stroke recurrence.

“You’re not immune to having another one […] Despite taking tablets to fluidify the blood” (Participant 21)

All of them, did not make the connection between adherence to treatment and lower risk of recurrence. For example, participant 8 reported that he missed information regarding risk of stroke recurrence and notably strategies he could implement to reduce his risk.

(speaking of the risk of recurrence) "I didn't have much information. Maybe it's missing." (Participant 8)

### Timeline

The notion of timeline appeared at various times in participants’ discourses. When defining stroke, some participants considered stroke to be an accident, whereas others considered it to be a chronic condition. The latter experienced difficulties in continuing their lives, went through a longer process of recovery, and perceived a higher risk of stroke recurrence.

“You know very well you’re not going to regain, er, all your, all your faculties, all the ones you had before, eh, you can imagine that, you can imagine that, yes, life is complicated afterward” (Participant 5)

Among participants who considered stroke as an accident (# 8, 11, 17, 19), there were two opposite profiles: those who thought they had recovered completely (a) and those for whom stroke was secondary compared to other comorbidities such as diabetes (# 17), cancer (#8, 11, 19) or peripheral artery disease (#11) (b).

(a) “For me the stroke was a matter of half an hour, eh. Because when I was in the neuro ward I’d recovered everything” (Participant 19)

(b) “You know, when you’ve also got cancer, I think that the more worrying of the two is the cancer, because in the medium or long term you know how it turns out, but, well, stroke is, I’d say, fleeting for a moment, mine was like this anyway. So, you know, it’s a bump in the road” (Participant 17)

### Emotional responses

Emotional responses were discussed for various post-stroke stages. Most participants reported no feeling of stress or anxiety at the acute phase. They reported a feeling of trust in the hospital services.

“I knew I was in safe hands, I wasn't worried. No, no, no, I wasn't worried.” (Participant 4)

At the chronic phase, most participants reported emotional difficulties post-stroke that strongly affected their daily life. They notably felt anxiety and fear regarding the risk of recurrence, and considered they had poor control over it.

“We don’t know, it can happen again. I was afraid it could. Oh yes, yes, there’s always this fear” (Participant 21)

They reported sadness about what they were experiencing.

“What saddens me is that as soon as I want to do something, I get tired too quickly” (Participant 12)

“I feel, how to say … humiliated by this … by this drop in … fitness. Thus I curl up on myself

” (Participant 13)

Participant 7 reported a sense of injustice, anger about the occurrence of stroke.

“We are not born under the same star [don’t have the same luck in life], there are people who do whatever they want and nothing happens to them, and there are others…” (Participant 7)

And participants 7, 10, 14, 16 reported having to cope with emotional responses they did not have to manage before.

“[since hospital discharge] I'm not feeling very well [emotionally]. I can't explain why” (Participant 7)

"emotions are much more intense […] you know, you cry for nothing […] I didn't have this kind of reactions before" (Participant 16)

Some other participants (1, 2, 3, 19, 20, 24) did not report notable emotional responses to stroke.

### Social support

Although not present in the CSM framework, the role of the social environment appeared predominant in participants’ discourse and was therefore included as an additional theme in our analysis. The social environment, especially the perceived support from friends, family and healthcare providers influenced other dimensions of the CSM. All participants reported that social support was a facilitator of managing stroke consequences, and lack of support was a barrier.

“if it weren't for them, I wouldn't be here either. I think I would have let myself wither in my opinion. It's a little bit my strength, my friends.” (Participant 18)

“If you have a family, if you have many people taking care of you, it's certainly less painful to manage all this […] when you're alone [like me] you can count only on yourself it's problematic, huh, it's problematic” (Participant 5).

At the acute phase, most participants (14 of 23) highlighted the importance of their relatives’ stroke beliefs who made the correct assessment and decision to contact emergency services.

“My wife quickly phoned our family doctor, who asked her to immediately call 15 (emergency medical services)” (Participant 19)

Relatives played also a role at the acute stage in rebuilding memories of what happened, and helping participants to construct their stroke identity, as mentioned in the identity section.

Participant 18 also mentioned the emphatic advice received from healthcare providers.

“[Healthcare providers] told me: your job is too stressful, you have to change your job, take care” (Participant 18)

At the chronic stage, participants (2, 6, 9, 10, 14, 20, 21, 24) who felt supported by their relatives and social network (colleagues…) implemented effective coping strategies and favourable health behaviors.

“colleagues, friend, family … I was really super well-supported really. Really well supported, it also pushes you to invest [in your recovery], not to let go, and to fight.” (Participant 9)

“I used to smoke 3 packets of cigarettes a day, well … stopping overnight is still … But I did it, well, I did it for my daughter.” (Participant 14)

Participant 9 also highlighted the importance of support from healthcare providers in the recovery process.

“[speaking of support from nurses] it's super important … It also pushes you to progress, to make efforts" (Participant 9)

Perceived support from friends, family, broader social network and healthcare providers seemed to influence motivation to recover, involvement in rehabilitation, and healthy behaviors.

## Discussion and conclusion (Table 3)

**Table 3:** Main findings and their link with implications for clinical practice and patient support interventions.

Main findings	Implications for clinical practice and patient support interventions
**Identity** Participants found it difficult to develop an illness identity around stroke experiences (lack of memories of the acute stage, difficulty to identify which impairments were related to stroke)	• Discuss symptoms/changes experienced during and after the acute stage and to what extent they could be linked to stroke or other factors • Consider carefully the timing for providing information: temporarily reduced capacity of the survivors to understand and memorize information at the acute phase may impair recall of information • Plan discussions to support persons rebuild memories of the acute stage and address denial or memory distortion
**Cause** Participants tended to minimize the contribution of behavioural risk factors	Discuss beliefs survivors have on stroke causes and provide relevant information about risk factors
**Consequences** Participants were autonomous in daily life but reported physical, psychological and cognitive consequences that induced participation restrictions	• Discuss consequences from the survivors’ perspectives • Assess the impact of stroke on social participation, reduce participation restrictions by considering modifiable factors that influence it
**Control** • Participants reported low perceived risk of recurrence, and regretted this was usually not discussed with healthcare providers • Beliefs of illness control reported by participants focused on medications and on the importance to train general population to recognize stroke symptoms	• Aware survivors of stroke and their relatives regarding stroke symptoms and the appropriate response to have in case of recurrence • Ask survivors how much control think they have on their illness and propose ways to enhance it (explanations regarding the mode of action of medication and advice concerning lifestyle and healthy behaviors)
**Timeline** Two profiles of participants were observed, those who considered stroke as an accident and those who considered it a chronic condition	• Asking persons with stroke about their own expectations concerning the duration of their condition may help align their perceptions with rehabilitation goals • Discuss beliefs about preventive behaviors patients have regarding stroke recurrence and plan appropriate support regarding their perception of timeline of stroke (action plans in short, medium or long-term)
**Emotional responses to stroke** • Resulting from both pathological and psychological process • At the chronic stage participants reported difficulty to manage unusual emotional responses (emotional lability, irritability, incessant crying) and frustration, sadness • A low sense of control regarding stroke recurrence generated fear and anxiety	Provide appropriate advice, resources, referral about the survivor’s specific emotional responses to stroke
**Social support** • Participants reported that social support influenced stroke cognitive beliefs, emotional responses and how easily they cope with stroke and its consequences • Participants found that perceived quality of patient-provider relationships influenced coping and adhering to recommendations	• Ask survivors’ satisfaction regarding their social support and propose solutions to enhance it (peer support group, stroke online forum…)Ask survivors’ satisfaction regarding their social support and propose solutions to enhance it (peer support group, stroke online forum…) • Train health professionals to listen to and take into account survivors’ beliefs about stroke and emotional responses to provide the most appropriate support

### Discussion

We found that even though participants experienced slight disability with a mRS ≤ 2, they reported difficulties in coping with the consequences of stroke, notably in relation to hidden disabilities.

Participants struggled to develop their illness identity beliefs, as they had poor or no memory at all of what happened at stroke onset and more globally during the acute phase. Santos et al. (2006) found that 41% of persons with stroke exhibited denial at the acute stage [28]. Beside physiological causes such as location of cerebral lesion (causing anosognosia) and presence of cognitive impairment, denial and distortion of memories of the traumatic event, called Memory Distortion for Traumatic Events [29], has also been described which may influence coping strategies [30,31] and recovery [29]. Irrespective of the mechanisms involved, it is possible that poor memory may have an impact on survivors’ coping and on their health behaviors. It also implies the timing for providing information about the condition and recommended lifestyle changes is crucial in promoting healthy behaviors and optimizing stroke self-management [32,33]. This suggests healthcare providers should be aware of the often reduced capability of the persons to understand and memorize information at the acute phase. Dedicated medical visits should be organized regularly over time to repeat information and ascertain it has been understood. As pointed out by one participant (#7), rebuilding his memories of what had happened with the help of his spouse was very helpful. Helping persons rebuild memories of the acute stage and identifying illness denial or memory distortion in persons with stroke could help them construct a stroke identity and find strategies to better cope with stroke at the chronic stage. It constitutes a step towards resilience and influences coping with stroke and its consequences [34]. Difficulties to form an illness identity were observed particularly in elderly participants with comorbidities who tended to attribute hidden disabilities to aging or comorbidities. It could be useful to provide survivors of stroke and providers with information on hidden disabilities to help persons with stroke implement strategies and subsequently strengthen their sense of control and self-management.

Participants agreed with healthcare providers’ causal attributions when they proposed medical causes such as atrial fibrillation or carotid stenosis but minimized the contribution of behavioral risk factors. This belief needs to be taken into account by healthcare professionals when counselling survivors of stroke on health behaviors needed to reduce cardiovascular risk factors.

Regarding perceived control, the risk of recurrence was usually not discussed with their healthcare providers and participants did not mention it explicitly. They mostly approached their low perceived control toward stroke recurrence indirectly by stressing the need of informing and training the general population to recognize stroke symptoms and the appropriate reactions. Consistently, beliefs of illness control reported by participants focused on medications mainly and healthy behaviors to a lesser extent. Regarding medication, most participants had very little knowledge about the mode of action and the expected benefit of their treatment, which contributed to their low perceived control regarding stroke recurrence. Regarding lifestyle and health behaviors, few participants received advice and they were not satisfied with information provided and pointed out the lack of support from healthcare providers. Perceived treatment control is a predictor of higher medication adherence in other conditions [19], this dimension should be addressed in specific educational programs providing information and support in behavior change particularly for persons who do not have access to rehabilitation services. In this way, several studies showed the effectiveness of community stroke educational groups [35,36]. Combining several media of information and educational support could improve survivors capability to use the information provided, as in nurse-led education programs [37], websites designed for this aim [36,38], case management [36], and peer-support groups [39].

Even though participants were mildly disabled, they reported significant consequences of stroke on their daily life. They spontaneously spoke about work and the impossibility of returning to their previous activities especially because of extreme fatigue and difficulty concentrating. Some participants perceived positive consequences of stroke. Analyzing perceived consequences from the survivor’s perspective in daily practice may help healthcare providers identify barriers and potential levers for recovery and find suitable solutions to improve satisfaction with recovery and prevent participation restrictions. These solutions could be included in community programs especially for persons with stroke who do not benefit from rehabilitation services. An example is the “Improving Participation After Stroke Self-Management Program (IPASS)”, a pilot program developed by an American team [40]. This community-based intervention designed for mild to moderate stroke (NHISS ≤ 16) consisted of a self-management education program in which survivors of stroke develop skills to self-manage, anticipate and find solutions regarding difficulties they may encounter in resuming valued activities. They showed that persons with mild to moderate stroke who had the IPASS intervention improved their self-efficacy to manage chronic stroke conditions and their participation in everyday life activities.

Regarding the Timeline dimension, two profiles of participants were observed, those who considered stroke as an accident and those who considered it a chronic condition. In our study, participants considering stroke as a long-lasting process experienced more difficulties in resuming valued activities and managing emotional responses. Asking persons with stroke about their own expectations concerning the duration of their condition may help align their perceptions with rehabilitation goals.

Emotional responses to stroke (emotional reactions caused by the stroke event) strongly affected daily life and some may be the results of post-stroke pathological and/or psychological processes. Emotional responses did not seem to be a problem at the acute stage where almost no feeling of stress or anxiety was reported. Conversely, at the chronic phase, difficulties regarding the management of unusual emotional responses were frequently reported notably emotional lability, irritability, incessant crying that impacted social relationships especially spousal relationships. Frustration and sadness were also pointed out. Low sense of control regarding recurrence generated fear and anxiety and some participants reported a feeling of unfairness. Emotional responses should be addressed by medical providers, in rehabilitation programs or through community intervention programs as persons with mild disabilities after stroke are often not eligible for rehabilitation services which are insufficient to accommodate all patients and are reserved primarily for the most disabled patients.

Additional to cognitive beliefs and emotional responses identified in the CSM, participants reported associations between social support, beliefs of stroke, and coping strategies. All reported participation restrictions due to stroke, but those who were well-supported and satisfied with their social interactions reported a lower burden of stroke in daily life. This is consistent with the positive impact of social support on stroke outcomes reported in the existing literature, especially with respect to social participation [41–44]. Moreover, the role of the social environment was highlighted by Leventhal et al. [45] as influencing illness beliefs, since social interactions are considered one of the three sources of information for developing illness representations, together with societal and cultural knowledge of an illness, and their own prior experience of illness. Thus, persons’ perceived satisfaction with their social support should be assessed by healthcare providers to anticipate difficulties in self-managing stroke and obstacles against healthy behavior adoption and help survivors who feel lonely or have less family support finding alternative forms of social support. Peer support groups showed their efficacy in improving satisfaction with social support perceived, notably through shared experiences and this was reported as essential to the recovery process [39]. Moreover, a previous study in England showed the advantages of a stroke online forum where persons with stroke and their families reported satisfaction with giving and receiving information, advice and social support [46].

The perceived quality of patient-provider relationships is also an important factor for coping and adhering to recommendations, partly through a better understanding of stroke and its physiological mechanisms. All participants were followed up by their general practitioner. This may be a good setting to discuss their beliefs and thus provide the explanations and information they need. Leventhal’s CSM proved useful, in an experimental study, for helping general practitioners discuss illness beliefs and action plans with patients and improving patients adherence to their medication regimen [47]. Several studies have shown that healthcare providers accurate understanding of their patients’ illness beliefs and needs had a positive impact on self-management. In a French study, general practitioners and persons with diabetes completed the IPQ-R questionnaire. Physicians were asked to estimate the most likely response of their patient. A better match between doctors’ and patients’ answers was associated with more effective illness self-management behaviors [48]. Self-management of survivors of stroke was found to be better when physicians had a better understanding of what survivors of stroke wanted to know about their condition and the way to manage it [48,49].

To our knowledge, this qualitative study is the first in-depth exploration of the illness beliefs of people suffering from mainly hidden disabilities after mild stroke. Our study also proposes an adapted Common-Sense Model of Self-Regulation after mild stroke which provides a comprehensive description of illness beliefs, emotional responses and the way persons cope with stroke and adopt favorable health behaviors. However, we acknowledge that this study presents some limitations. Qualitative research is characterized by inherent subjectivity, which we tried to limit by discussing the results of analysis with all members of our interdisciplinary team to obtain consensus of interpretation. It would be useful to replicate this research in other settings to see whether cultural differences may arise, particularly in terms of social support. Furthermore, the type of disability may have also an impact on results, therefore replication on different groups of survivors of stroke would be certainly informative. Additionally, quantitative studies using the Illness Perception Questionnaire [15], a validated tool widely used in other chronic illnesses [50], could be performed and confronted with qualitative data using mixed methods approaches in order to maximize relevant information on stroke cognitive beliefs and emotional responses and build a solid foundation for intervention development.

### Conclusion

The study revealed key elements within the CSM framework for a better understanding of stroke beliefs and coping strategies in survivors with a mRS ≤ 2 and mostly with hidden disability. These elements were the difficulties constructing an illness identity at acute stage mainly due to cognitive impairment and recognizing hidden disabilities as stroke consequences at the chronic phase. Non-acceptance of behavioral risk factors as causes of stroke was associated with a lack or inappropriate timing of information from healthcare providers regarding risk factors and medications and their impact on stroke recurrence risk, and led to poor perceived control of stroke recurrence which generated anxiety, fear, and loss of self-confidence. Perceived support from relatives, broader social networks and healthcare providers influenced the way persons with stroke get involved in the recovery process and adopt healthy behaviors. Healthcare providers attention to individuals’ perception of their stroke timeline may improve coping. Survivors of stroke with mild-no disability still may have social participation restrictions and these should be addressed in medical follow-up. Coping with stroke and consequences at the chronic stage generated in most cases emotional responses such as fear, anxiety, frustration, or unusual emotional reactions difficult to manage, that should be addressed in clinical practice.

This study provides new insights on how participants give meaning to their stroke and on potential facilitators and barriers regarding favorable health behaviors. There is a need to consider cognitive beliefs and emotional responses of survivors of stroke in clinical practice, to help them manage consequences of stroke in daily life, develop problem-solving strategies, and adopt favorable behaviors.

## Supporting information

S1 Table Consolidated criteria for reporting qualitative studies (COREQ): 32-item checklist. (DOC)
